# TbNb_6_Sn_6_: the first ternary compound from the rare earth–niobium–tin system

**DOI:** 10.1107/S1600536810045964

**Published:** 2010-11-17

**Authors:** Igor Oshchapovsky, Volodymyr Pavlyuk, Thomas F. Fässler, Viktor Hlukhyy

**Affiliations:** a Department of Inorganic Chemistry, Ivan Franko Lviv National University, Kyryla and Mefodiya str. 6, 79005 Lviv, Ukraine; b Department Chemie, Technische Universität München, Lichtenbergstr. 4, 85747 Garching, Germany

## Abstract

The title compound, terbium hexa­niobium hexastannide, TbNb_6_Sn_6_, is the first ternary compound from the rare earth–niobium–tin system. It has the HfFe_6_Ge_6_ structure type, which can be analysed as an inter­growth of the Zr_4_Al_3_ and CaCu_5_ structures. All the atoms lie on special positions; their coordination geometries and site symmetries are: Tb (dodeca­hedron) 6/*mmm*; Nb (distorted icosa­hedron) 2*mm*; Sn (Frank–Caspar polyhedron, CN = 14–15) 6*mm* and 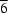 *m*2; Sn (distorted icosa­hedron) 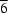 *m*2. The structure contains a graphite-type Sn network, Kagome nets of Nb atoms, and Tb atoms alternating with Sn2 dumbbells in the channels.

## Related literature

For background to niobium alloys, see: Ateev & Shamrai (1966 ▶). For related structures and background to inter­metallics, see: Nowotny (1942 ▶); Raeuchle & Rundle (1952 ▶); Schobinger-Papamantellos *et al.* (1998 ▶); Wilson *et al.* (1960 ▶). A statistical test of the distribution of the *E* values using the program *E-STATS* from *WinGX* system (Farrugia, 1999 ▶) suggested that the structure is centrosymmetric. For MgCo_6_Ge_6_, see: Gieck *et al.* (2006 ▶).

## Experimental

### 

#### Crystal data

TbNb_6_Sn_6_
*M* *_r_* = 1428.52
Hexagonal, 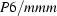

*a* = 5.7650 (1) Å
*c* = 9.5387 (4) Å
*V* = 274.55 (1) Å^3^
*Z* = 1
Mo *K*α radiation
μ = 25.66 mm^−1^
*T* = 293 K
0.050 × 0.020 × 0.004 mm

#### Data collection

Oxford Diffraction Xcalibur3 diffractometer with CCD detector
Absorption correction: multi-scan (*CrysAlis RED*; Oxford Diffraction, 2008 ▶) *T*_min_ = 0.592, *T*_max_ = 1.000
2493 measured reflections
208 independent reflections
181 reflections with *I* > 2σ(*I*)
*R*_int_ = 0.027

#### Refinement

*R*[*F* ^2^ > 2σ(*F* ^2^)] = 0.017
*wR*(*F* ^2^) = 0.038
*S* = 1.29
208 reflections
15 parameters
Δρ_max_ = 1.68 e Å^−3^
Δρ_min_ = −2.35 e Å^−3^

### 

Data collection: *CrysAlis CCD* (Oxford Diffraction, 2008 ▶); cell refinement: *CrysAlis CCD*; data reduction: *CrysAlis RED* (Oxford Diffraction, 2008 ▶); program(s) used to solve structure: *SHELXS97* (Sheldrick, 2008 ▶); program(s) used to refine structure: *SHELXL97* (Sheldrick, 2008 ▶); molecular graphics: *DIAMOND* (Brandenburg, 2006 ▶); software used to prepare material for publication: *publCIF* (Westrip, 2010 ▶).

## Supplementary Material

Crystal structure: contains datablocks I, global. DOI: 10.1107/S1600536810045964/hb5709sup1.cif

Structure factors: contains datablocks I. DOI: 10.1107/S1600536810045964/hb5709Isup2.hkl

Additional supplementary materials:  crystallographic information; 3D view; checkCIF report

## Figures and Tables

**Fig. 1.**
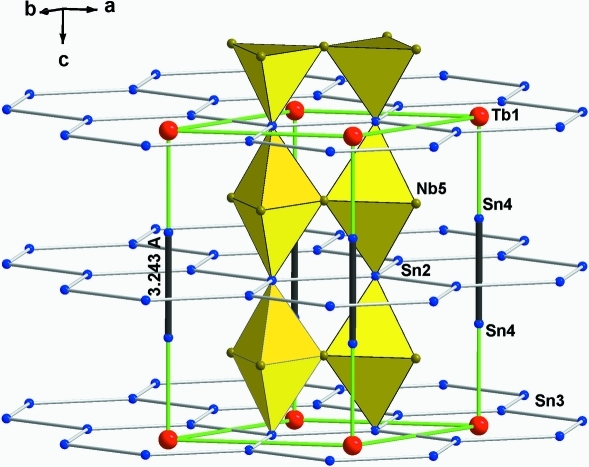
View of the TbNb6Sn6 structure. The graphite-type Sn layers, Ge2 dumbbells and condensed Nb3Sn2 bipyramids are emphasized.

**Fig. 2.**
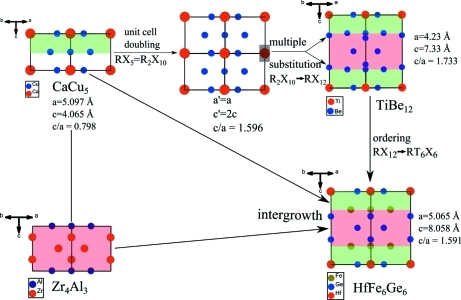
Relationships between the HfFe6Ge6, Zr4Al3 and CaCu5 structures.

## supplementary crystallographic information

### Comment

Niobium compounds have very useful properties for superconductive materials: high critical fields and plasticity which gives an opportunity to make superconductive windings. The main disadvantage of these compounds is the low critical temperature (Ateev & Shamrai, 1966). Superconductive Nb_3_Sn with T_c_=18.2 K is a high performance superconductor and the gold standard of the world's superconductor industry.

So far, no ternary compounds in the RE–Nb–Sn (RE - rare-earth metals) systems are known. TbNb_6_Sn_6_ is the first ternary Rare earth – Niobium – Tin compound. According to the X-ray single-crystal data the TbNb_6_Sn_6_ compound crystallizes with hexagonal symmetry (space group *P*6/*mmm*, HfFe_6_Ge_6_ structure type). In TbNb_6_Sn_6_ planar graphite-type layers of Sn atoms and Kagome nets of Nb atoms alternate along the *c* axis, similar to the recently reported MgCo_6_Ge_6_ compound (Gieck *et al.*, 2006). The resulting columns of vertex-sharing trigonal bipyramids form a three-dimensional Nb–Sn network with hexagonal tunnels (Figure 1). These tunnels are alternately centered by Tb atoms and Sn2 dumbbells (with Sn-Sn distances of 3.24 A). The coordination polyhedra of the atoms are: Tb1 — 20-vertex polyhedron (CN=20), Sn2 — Frank-Casper polyhedron (CN=15), Sn3 — distorted icosahedron (CN=12), Sn4 — Frank-Casper polyhedron (CN=14) and Nb5 — distorted icosahedron (CN=12).

The HfFe_6_Ge_6_ structure type (Schobinger-Papamantellos *et al.*,1998), also referred to MgFe_6_Ge_6_ or LiCo_6_Ge_6_, can be described as an intergrowth of the Zr_4_Al_3_ (Wilson *et al.*, 1960) and CaCu_5_ (Nowotny, 1942) structure types (see Fig.2). Another possibility to describe the HfFe_6_Ge_6_ structure is a transformation of the CaCu_5_ structure via multiple substitution and ordering of atoms. The first step is the doubling of the CaCu_5_ unit cell along the *c* axis. The substitution of every second Ca atom (R) along the *c* axis by a pair of atoms (2X) transforms this structure into the hexagonal modification of TiBe_12_ (Raeuchle & Rundle, 1952). As a result, the *c/a* ratio increases to 1.733. Further ordering of X atoms in the TiBe_12_ leads to the HfFe_6_Ge_6_ structure. Perhaps the presence of atoms of different radii leads to closer packing of the layers in the ternary HfFe_6_Ge_6_ compared to the binary TiBe_12_. Therefore, the c/a ratio of 1.591 for ternary HfFe_6_Ge_6_ is much closer to the ideal value of 1.596 (*c/a* = 1.6546 for TbNb_6_Sn_6_).

### Experimental

Single crystals of the title compound were first found in a sample with the composition 2Tb:3Zn:5Sn, which was synthesized by induction heating of the pure elements in a niobium crucible. The sample was heated at 1100° C in an induction furnace (Hüttinger Elektronik, Freiburg, Type TIG 2.5/300) under continuous argon flow for 1 h followed by cooling to 700° C at a rate of 10 degrees/min. Finally it was quenched by switching off the furnace. A reaction between the sample and the Nb container was observed. Good-quality hexagonal plate-like crystals were selected from annealed sample by mechanical fragmentation. Single-crystal intensity data of TbNb_6_Sn_6_ were collected at room temperature on an Oxford-Xcalibur3 CCD area detector diffractometer. After the measurement, the single crystal was analyzed with a JEOL SEM 5900LV scanning electron microscope. No impurity elements heavier than sodium were observed. The EDX analysis of well-shaped single crystal reveals the composition (in atomic  percentages) Tb 8(3), Nb 45 (6), and Sn 47 (7), which is in good agreement with  the compositions resulting from XRD data refinement. Further, a sample with the composition of Tb:6Nb:6Sn was prepared by arc melting and examined by powder X-ray diffraction. As-cast sample does not contain TbNb_6_Sn_6_ phase. However, after grinding, pressing and annealing it at 900°C for 12 h a significant amount of the TbNb_6_Sn_6 _phase was observed. Magnetic measurements of the annealed sample were performed using a MPMS XL5 SQUID magnetometer (Quantum Design). Cooling the sample without magnetic field and rising the temperature in the presence of a field of 15 Oe from 1.8 K to 50 K revealed at approximately 18 K only the superconducting behavior of the Nb_3_Sn impurity.

### Refinement

A statistical test of the distribution of the E values using the program E-STATS from *WinGX* system (Farrugia, 1999) suggested that the structure is centrosymmetric. The analysis of systematic extinctions yielded the space group *P6/mmm*, and it was confirmed by the following structure refinement. The structure was solved by the direct methods.

### Crystal data

**Table:**

TbNb_6_Sn_6_	*D*_x_ = 8.640 Mg m^−^^3^
*M**_r_* = 1428.52	Mo *K*α radiation, λ = 0.71073 Å
Hexagonal, *P*6/*m**m**m*	Cell parameters from 181 reflections
Hall symbol: -P 6 2	θ = 4.1–30.0°
*a* = 5.7650 (1) Å	µ = 25.66 mm^−^^1^
*c* = 9.5387 (4) Å	*T* = 293 K
*V* = 274.55 (1) Å^3^	Hexagonal plate, metallic gray
*Z* = 1	0.05 × 0.02 × 0.004 mm
*F*(000) = 611	

### Data collection

**Table:**

Oxford Diffraction Xcalibur3 diffractometer with CCD detector	208 independent reflections
Radiation source: Enhance (Mo) X-ray Source	181 reflections with *I* > 2σ(*I*)
graphite	*R*_int_ = 0.027
Detector resolution: 16.0238 pixels mm^-1^	θ_max_ = 30.0°, θ_min_ = 4.1°
ω and π scans	*h* = −8→7
Absorption correction: multi-scan (*CrysAlis RED*, Oxford Diffraction, 2008)	*k* = −5→8
*T*_min_ = 0.592, *T*_max_ = 1.000	*l* = −13→13
2493 measured reflections	

### Refinement

**Table:**

Refinement on *F*^2^	Primary atom site location: structure-invariant direct methods
Least-squares matrix: full	Secondary atom site location: difference Fourier map
*R*[*F*^2^ > 2σ(*F*^2^)] = 0.017	*w* = 1/[σ^2^(*F*_o_^2^) + (0.0113*P*)^2^ + 2.3749*P*] where *P* = (*F*_o_^2^ + 2*F*_c_^2^)/3
*wR*(*F*^2^) = 0.038	(Δ/σ)_max_ < 0.001
*S* = 1.29	Δρ_max_ = 1.68 e Å^−^^3^
208 reflections	Δρ_min_ = −2.35 e Å^−^^3^
15 parameters	Extinction correction: *SHELXL97* (Sheldrick, 2008), Fc^*^=kFc[1+0.001xFc^2^λ^3^/sin(2θ)]^-1/4^
0 restraints	Extinction coefficient: 0.0333 (12)

### Special details

**Table:**

Geometry. All e.s.d.'s (except the e.s.d. in the dihedral angle between two l.s. planes) are estimated using the full covariance matrix. The cell e.s.d.'s are taken into account individually in the estimation of e.s.d.'s in distances, angles and torsion angles; correlations between e.s.d.'s in cell parameters are only used when they are defined by crystal symmetry. An approximate (isotropic) treatment of cell e.s.d.'s is used for estimating e.s.d.'s involving l.s. planes.
Refinement. Refinement of *F*^2^ against ALL reflections. The weighted *R*-factor *wR* and goodness of fit *S* are based on *F*^2^, conventional *R*-factors *R* are based on *F*, with *F* set to zero for negative *F*^2^. The threshold expression of *F*^2^ > σ(*F*^2^) is used only for calculating *R*-factors(gt) *etc*. and is not relevant to the choice of reflections for refinement. *R*-factors based on *F*^2^ are statistically about twice as large as those based on *F*, and *R*- factors based on ALL data will be even larger.

### Fractional atomic coordinates and isotropic or equivalent isotropic displacement parameters (Å^2^)

**Table:**

	*x*	*y*	*z*	*U*_iso_*/*U*_eq_	
Tb1	0.0000	0.0000	0.0000	0.0101 (3)	
Sn2	0.3333	0.6667	0.5000	0.0074 (2)	
Sn3	0.3333	0.6667	0.0000	0.0066 (2)	
Sn4	0.0000	0.0000	0.33003 (11)	0.0091 (2)	
Nb5	0.0000	0.5000	0.24932 (6)	0.0053 (2)	

### Atomic displacement parameters (Å^2^)

**Table:**

	*U*^11^	*U*^22^	*U*^33^	*U*^12^	*U*^13^	*U*^23^
Tb1	0.0082 (3)	0.0082 (3)	0.0140 (5)	0.00408 (16)	0.000	0.000
Sn2	0.0084 (3)	0.0084 (3)	0.0054 (4)	0.00421 (14)	0.000	0.000
Sn3	0.0070 (3)	0.0070 (3)	0.0058 (4)	0.00349 (14)	0.000	0.000
Sn4	0.0063 (3)	0.0063 (3)	0.0148 (5)	0.00314 (15)	0.000	0.000
Nb5	0.0047 (3)	0.0050 (3)	0.0060 (3)	0.00235 (17)	0.000	0.000

### Geometric parameters (Å, °)

**Table:**

Tb1—Sn4	3.1481 (10)	Sn3—Nb5^ix^	2.9027 (5)
Tb1—Sn4^i^	3.1481 (10)	Sn3—Nb5	2.9027 (5)
Tb1—Sn3^ii^	3.3284	Sn3—Tb1^xiii^	3.3284
Tb1—Sn3^iii^	3.3284	Sn3—Tb1^xiv^	3.3284
Tb1—Sn3^i^	3.3284	Sn4—Nb5	2.9835 (3)
Tb1—Sn3	3.3284	Sn4—Nb5^xv^	2.9835 (3)
Tb1—Sn3^iv^	3.3285	Sn4—Nb5^ix^	2.9835 (3)
Tb1—Sn3^v^	3.3285	Sn4—Nb5^xvi^	2.9835 (3)
Sn2—Nb5	2.9133 (5)	Sn4—Nb5^v^	2.9835 (3)
Sn2—Nb5^vi^	2.9133 (5)	Sn4—Nb5^xvii^	2.9835 (3)
Sn2—Nb5^vii^	2.9133 (5)	Sn4—Sn4^xviii^	3.243 (2)
Sn2—Nb5^viii^	2.9133 (5)	Nb5—Nb5^viii^	2.8825
Sn2—Nb5^ix^	2.9133 (5)	Nb5—Nb5^xvi^	2.8825
Sn2—Nb5^x^	2.9133 (5)	Nb5—Nb5^ix^	2.8825
Sn3—Nb5^viii^	2.9027 (5)	Nb5—Nb5^xix^	2.8825
Sn3—Nb5^iv^	2.9027 (5)	Nb5—Sn3^iv^	2.9027 (5)
Sn3—Nb5^xi^	2.9027 (5)	Nb5—Sn2^vii^	2.9133 (5)
Sn3—Nb5^xii^	2.9027 (5)	Nb5—Sn4^xiii^	2.9835 (3)
			
Sn4—Tb1—Sn4^i^	180.0	Nb5—Sn3—Tb1	73.341 (3)
Sn4—Tb1—Sn3^ii^	90.0	Tb1^xiii^—Sn3—Tb1	120.0
Sn4^i^—Tb1—Sn3^ii^	90.0	Tb1^xiv^—Sn3—Tb1	120.0
Sn4—Tb1—Sn3^iii^	90.0	Nb5—Sn4—Nb5^xv^	113.586 (17)
Sn4^i^—Tb1—Sn3^iii^	90.0	Nb5—Sn4—Nb5^ix^	57.772 (6)
Sn3^ii^—Tb1—Sn3^iii^	180.0	Nb5^xv^—Sn4—Nb5^ix^	150.09 (4)
Sn4—Tb1—Sn3^i^	90.0	Nb5—Sn4—Nb5^xvi^	57.772 (6)
Sn4^i^—Tb1—Sn3^i^	90.0	Nb5^xv^—Sn4—Nb5^xvi^	57.772 (6)
Sn3^ii^—Tb1—Sn3^i^	120.0	Nb5^ix^—Sn4—Nb5^xvi^	113.586 (17)
Sn3^iii^—Tb1—Sn3^i^	60.0	Nb5—Sn4—Nb5^v^	150.09 (4)
Sn4—Tb1—Sn3	90.0	Nb5^xv^—Sn4—Nb5^v^	57.772 (6)
Sn4^i^—Tb1—Sn3	90.0	Nb5^ix^—Sn4—Nb5^v^	113.586 (17)
Sn3^ii^—Tb1—Sn3	60.0	Nb5^xvi^—Sn4—Nb5^v^	113.586 (17)
Sn3^iii^—Tb1—Sn3	120.0	Nb5—Sn4—Nb5^xvii^	113.586 (17)
Sn3^i^—Tb1—Sn3	180.0	Nb5^xv^—Sn4—Nb5^xvii^	113.586 (17)
Sn4—Tb1—Sn3^iv^	90.0	Nb5^ix^—Sn4—Nb5^xvii^	57.772 (6)
Sn4^i^—Tb1—Sn3^iv^	90.0	Nb5^xvi^—Sn4—Nb5^xvii^	150.09 (4)
Sn3^ii^—Tb1—Sn3^iv^	120.0	Nb5^v^—Sn4—Nb5^xvii^	57.772 (6)
Sn3^iii^—Tb1—Sn3^iv^	60.0	Nb5—Sn4—Tb1	75.05 (2)
Sn3^i^—Tb1—Sn3^iv^	120.0	Nb5^xv^—Sn4—Tb1	75.05 (2)
Sn3—Tb1—Sn3^iv^	60.0	Nb5^ix^—Sn4—Tb1	75.05 (2)
Sn4—Tb1—Sn3^v^	90.0	Nb5^xvi^—Sn4—Tb1	75.05 (2)
Sn4^i^—Tb1—Sn3^v^	90.0	Nb5^v^—Sn4—Tb1	75.05 (2)
Sn3^ii^—Tb1—Sn3^v^	60.0	Nb5^xvii^—Sn4—Tb1	75.05 (2)
Sn3^iii^—Tb1—Sn3^v^	120.0	Nb5—Sn4—Sn4^xviii^	104.95 (2)
Sn3^i^—Tb1—Sn3^v^	60.0	Nb5^xv^—Sn4—Sn4^xviii^	104.95 (2)
Sn3—Tb1—Sn3^v^	120.0	Nb5^ix^—Sn4—Sn4^xviii^	104.95 (2)
Sn3^iv^—Tb1—Sn3^v^	180.0	Nb5^xvi^—Sn4—Sn4^xviii^	104.95 (2)
Nb5—Sn2—Nb5^vi^	146.807 (6)	Nb5^v^—Sn4—Sn4^xviii^	104.95 (2)
Nb5—Sn2—Nb5^vii^	110.325 (14)	Nb5^xvii^—Sn4—Sn4^xviii^	104.95 (2)
Nb5^vi^—Sn2—Nb5^vii^	59.302 (11)	Tb1—Sn4—Sn4^xviii^	180.0
Nb5—Sn2—Nb5^viii^	59.302 (11)	Nb5^viii^—Nb5—Nb5^xvi^	180.0
Nb5^vi^—Sn2—Nb5^viii^	110.325 (14)	Nb5^viii^—Nb5—Nb5^ix^	60.0
Nb5^vii^—Sn2—Nb5^viii^	146.808 (6)	Nb5^xvi^—Nb5—Nb5^ix^	120.0
Nb5—Sn2—Nb5^ix^	59.302 (11)	Nb5^viii^—Nb5—Nb5^xix^	120.0
Nb5^vi^—Sn2—Nb5^ix^	146.808 (6)	Nb5^xvi^—Nb5—Nb5^xix^	60.0
Nb5^vii^—Sn2—Nb5^ix^	146.808 (6)	Nb5^ix^—Nb5—Nb5^xix^	180.0
Nb5^viii^—Sn2—Nb5^ix^	59.302 (11)	Nb5^viii^—Nb5—Sn3	60.229 (6)
Nb5—Sn2—Nb5^x^	146.807 (6)	Nb5^xvi^—Nb5—Sn3	119.770 (6)
Nb5^vi^—Sn2—Nb5^x^	59.302 (11)	Nb5^ix^—Nb5—Sn3	60.229 (6)
Nb5^vii^—Sn2—Nb5^x^	59.302 (11)	Nb5^xix^—Nb5—Sn3	119.770 (6)
Nb5^viii^—Sn2—Nb5^x^	146.808 (6)	Nb5^viii^—Nb5—Sn3^iv^	119.770 (6)
Nb5^ix^—Sn2—Nb5^x^	110.325 (14)	Nb5^xvi^—Nb5—Sn3^iv^	60.229 (6)
Nb5^viii^—Sn3—Nb5^iv^	146.683 (6)	Nb5^ix^—Nb5—Sn3^iv^	119.771 (6)
Nb5^viii^—Sn3—Nb5^xi^	110.033 (14)	Nb5^xix^—Nb5—Sn3^iv^	60.229 (6)
Nb5^iv^—Sn3—Nb5^xi^	59.541 (11)	Sn3—Nb5—Sn3^iv^	69.967 (14)
Nb5^viii^—Sn3—Nb5^xii^	146.683 (6)	Nb5^viii^—Nb5—Sn2	60.349 (6)
Nb5^iv^—Sn3—Nb5^xii^	59.541 (12)	Nb5^xvi^—Nb5—Sn2	119.652 (6)
Nb5^xi^—Sn3—Nb5^xii^	59.541 (11)	Nb5^ix^—Nb5—Sn2	60.349 (6)
Nb5^viii^—Sn3—Nb5^ix^	59.541 (11)	Nb5^xix^—Nb5—Sn2	119.652 (6)
Nb5^iv^—Sn3—Nb5^ix^	146.683 (6)	Sn3^iv^—Nb5—Sn2	179.854 (14)
Nb5^xi^—Sn3—Nb5^ix^	146.683 (6)	Nb5^viii^—Nb5—Sn2^vii^	119.651 (6)
Nb5^xii^—Sn3—Nb5^ix^	110.033 (14)	Nb5^xvi^—Nb5—Sn2^vii^	60.349 (6)
Nb5^viii^—Sn3—Nb5	59.541 (12)	Nb5^ix^—Nb5—Sn2^vii^	119.652 (6)
Nb5^iv^—Sn3—Nb5	110.033 (14)	Nb5^xix^—Nb5—Sn2^vii^	60.349 (6)
Nb5^xi^—Sn3—Nb5	146.683 (6)	Sn3—Nb5—Sn2^vii^	179.855 (14)
Nb5^xii^—Sn3—Nb5	146.683 (6)	Sn3^iv^—Nb5—Sn2^vii^	110.179 (2)
Nb5^ix^—Sn3—Nb5	59.541 (11)	Sn2—Nb5—Sn2^vii^	69.676 (14)
Nb5^viii^—Sn3—Tb1^xiii^	73.341 (3)	Nb5^viii^—Nb5—Sn4	118.886 (3)
Nb5^iv^—Sn3—Tb1^xiii^	73.341 (3)	Nb5^xvi^—Nb5—Sn4	61.114 (3)
Nb5^xi^—Sn3—Tb1^xiii^	73.341 (3)	Nb5^ix^—Nb5—Sn4	61.114 (3)
Nb5^xii^—Sn3—Tb1^xiii^	124.984 (7)	Nb5^xix^—Nb5—Sn4	118.886 (3)
Nb5^ix^—Sn3—Tb1^xiii^	124.984 (7)	Sn3—Nb5—Sn4	102.205 (16)
Nb5—Sn3—Tb1^xiii^	73.341 (3)	Sn3^iv^—Nb5—Sn4	102.205 (17)
Nb5^viii^—Sn3—Tb1^xiv^	73.341 (3)	Sn2—Nb5—Sn4	77.773 (17)
Nb5^iv^—Sn3—Tb1^xiv^	124.984 (7)	Sn2^vii^—Nb5—Sn4	77.773 (17)
Nb5^xi^—Sn3—Tb1^xiv^	73.341 (3)	Nb5^viii^—Nb5—Sn4^xiii^	61.114 (3)
Nb5^xii^—Sn3—Tb1^xiv^	73.341 (3)	Nb5^xvi^—Nb5—Sn4^xiii^	118.886 (3)
Nb5^ix^—Sn3—Tb1^xiv^	73.341 (3)	Nb5^ix^—Nb5—Sn4^xiii^	118.886 (3)
Nb5—Sn3—Tb1^xiv^	124.983 (7)	Nb5^xix^—Nb5—Sn4^xiii^	61.114 (3)
Tb1^xiii^—Sn3—Tb1^xiv^	120.0	Sn3—Nb5—Sn4^xiii^	102.205 (17)
Nb5^viii^—Sn3—Tb1	124.984 (7)	Sn3^iv^—Nb5—Sn4^xiii^	102.205 (16)
Nb5^iv^—Sn3—Tb1	73.341 (3)	Sn2—Nb5—Sn4^xiii^	77.773 (17)
Nb5^xi^—Sn3—Tb1	124.983 (7)	Sn2^vii^—Nb5—Sn4^xiii^	77.772 (17)
Nb5^xii^—Sn3—Tb1	73.341 (3)	Sn4—Nb5—Sn4^xiii^	150.09 (4)
Nb5^ix^—Sn3—Tb1	73.341 (3)		

Symmetry codes: (i) −*x*, −*y*, −*z*; (ii) −*x*+1, −*y*+1, −*z*; (iii) *x*−1, *y*−1, *z*; (iv) −*x*, −*y*+1, −*z*; (v) *x*, *y*−1, *z*; (vi) *x*−*y*+1, *x*+1, −*z*+1; (vii) −*x*, −*y*+1, −*z*+1; (viii) −*x*+*y*, −*x*+1, *z*; (ix) −*y*+1, *x*−*y*+1, *z*; (x) *y*, −*x*+*y*, −*z*+1; (xi) *x*−*y*+1, *x*+1, −*z*; (xii) *y*, −*x*+*y*, −*z*; (xiii) *x*, *y*+1, *z*; (xiv) *x*+1, *y*+1, *z*; (xv) −*y*, *x*−*y*, *z*; (xvi) −*x*+*y*−1, −*x*, *z*; (xvii) −*x*+*y*, −*x*, *z*; (xviii) −*x*, −*y*, −*z*+1; (xix) −*y*, *x*−*y*+1, *z*.
